# On Baths and Mineral Waters

**Published:** 1832

**Authors:** 


ART. VI.—*On Baths and Mineral Waters.* In two parts. Part I. A full account of the hygienic and curative powers of *cold*, *tepid*, *warm*, *hot*, and *vapor* baths, and of *sea* bathing. Part II. A history of the chemical composition, and medicinal properties of the chief Mineral Springs of the United States and of Europe. By JOHN BELL, M. D., Lecturer on the Institutes of Medicine and Medical Jurisprudence, &c. &c. Philadelphia, Carey & Lea.

The subject of the present volume, although one of great importance to the physician, and one upon which his advice is frequently asked, is scarcely thought of as a necessary or important part of his education. Believing the treatise of Dr. Bell to be well calculated to draw the attention of physicians to this neglected department of their profession, we shall notice a few of the most important of the topics upon which he treats, for the purpose rather of exciting than of allaying the curiosity of our readers.

Chapter I. is devoted to the history of Bathing, among different nations and in different ages of the world. Every person is familiar with the estimation in which Bathing was held among the ancient Greeks and Romans, and with the eagerness with which it is resorted to by the Eastern and Northern nations of the present day. This attachment has laid the foundation for a species of luxury, in which the utmost extravagance is manifested; of this the author has collected many curious instances in the present volume.

Chapter II. is devoted to the skin, its anatomical and physiological properties, its sympathies, &c. The following is a summary of the principles he advances:

"1. The skin, with a superficies in an adult of about fifteen square feet, consists of three laminæ, the outer one hard, and in a measure horny; the second and middle soft and pulpy; the third and inner firm and resisting, with a great many fine vessels and nerves spread over it after having penetrated it from the fatty and cellular tissue beneath.

2. From the cutaneous surface is secreted and exhaled different gasses, the chief of which is carbonic acid gas or fixed air, a watery vapor or insensible perspiration, and at times an aqueous fluid, holding salts in solution, and constituting what is called sweat: from small bodies or glands there is also discharged on particular regions of the skin, an oleaginous fluid. The skin absorbs or allows to pass through its pores various gasses, the chief of which is oxygen gas, and also watery vapor.

3. In addition to its other offices of supply and discharge, the skin is also the seat of the sense of touch, and as such is connected with the brain, and other senses, by a variety of relations.

4. Acting by a well established law in physics, the skin is a surface for evaporation and consequent cooling.

5. This tissue is continuous in the various openings at the eyes, nostrils, lips, &c. with the membranes lining the eye-lids, and those on which take place the senses of smell and taste; also with those continued from these parts down into the lungs in one direction, and stomach and intestines in another, and on which take place the chief phenomena of respiration, and of the different stages of digestion. In another direction, the skin is continuous with the mucous membranes lining the organs peculiar to each sex.

6. The sympathies of the cutaneous tissue with these internal membranes, must, *a priori*, seem evident, as well from the general sameness of structure as from community of office—the skin exhaling and absorbing vapors and gasses like the lungs, and fluids like the digestive canal. But not only does it sympathise with these, but with other membranes of the serous class, such as that investing the lungs, and the one covering the abdominal viscera; also, that variety lining the joints, &c.

7. The influence of the skin over the animal economy must be manifestly great, and of course the operation of the agents, by which it is impressed, ought to be familiar to us. These act in two ways. 1. By exciting or diminishing its nutritive functions of absorption, and secretion and excretion; and, 2. By increasing or diminishing its sensibility and sensations.

Bathing affects it in all these ways: by softening the cuticle or outer hard lamina of the skin, a more ready entrance is afforded to water and other fluids, and by subsequent friction, this part more readily peels off, so as to leave the skin more delicate for the discharge of its various functions: in addition to this, baths, by their temperature acting on their sentient extremities of the nerves, and the minute blood-vessels blended with them, will either accelerate or retard the functions, according as the medium in which the body is immersed be hot or cold.

8. Simultaneously with these changes in the functions of the skin, or immediately consequent upon them, will be changes in the nervous system in general, and in the circulation, respiration, digestion, and various secretions, by the links and means already designated.

9. The effects of bathing are relative to the temperature of the fluid and that of the living body to which it is applied.

10. The medium temperature of the human body is from 96 to 98 deg. Fahrenheit. The warmth is less at the extremities than in the central parts of the body, so that in this respect there is often a difference of 6 or 8 deg. between the hands and the arm pit, even when the former do not feel at all cold to their possessor.

11. Although our bodies are, in degree, obedient to the laws which govern the transmission of caloric in the material world generally, yet we have, obviously, independent sources of heat, and means for preserving an equilibrium of the animal temperature.

12. The air in which we live being almost all of a lower temperature than that of the body, there is a constant evolution, and discharge of caloric from the latter into the former. This loss would become excessive in low temperatures, but for the covering of the skin, which is a bad conductor, and the protection afforded by clothing of woollen, or furs, which are also imperfect conductors of heat.

13. In the winter season, and in cold climates, the digestion is more vigorous, respiration fuller and complete, and nutrition more active; there is, in fine, an activity of all the organs of internal life, by which caloric is secreted. The absence, in a great measure of sensible perspiration, or sweat, prevents the loss of heat by evaporation, and the skin from being such a good conductor as in the opposite state of things.

14. The excitement produced by living in air of an elevated temperature, is moderated by the increased cutaneous secretion, or sweat, and consequent evaporation.

15. Our sensations of heat will mainly depend on the proportion between the formation of caloric in the organs of the animal economy, and its expenditure by the surrounding air and inert substances with which we are in contact.

16. Habit reconciles us to a wide range of atmospherical temperature. Cold air, unless so extreme as to benumb the nervous expansions of the skin, and enfeeble the nervous energy and functions generally, and hot air, equal to, and sometimes exceeding the medium temperature of the body, unless perspiration be suppressed, are both well borne. In both the average heat of the body remains the same.

17. The exhausting effects of cold, always readily felt at the extremities of the body, must be obviated, by attention to all the means, already indicated, for giving energy to the system, or in other words, increasing the action of the minute vessels and nerves of the organs, by which caloric is evolved.

18. The more vigorous the circulation and nervous energy, the better can cold be borne. External heat, then, by exciting the functions, and contributing to the evolution of animal heat, enables us the better to resist cold. This must be understood of the equable operation of external heat, and its not being continued so long as to exhaust the minute vessels of the skin by copious perspiration, and of other parts by abundant discharges.

19. The medium temperature of the body is less in infancy and old age, and the ability to resist at these times the exhausting effects of cold is also less.

20. Inordinate heat of the body is felt when the cooling processes, by perspiration and evaporation, are checked. It then becomes necessary to moderate the excessive action of the organs of the body, from all of which the caloric is evolved. This is best done by cold applied to the skin, or by means of cold drinks to the stomach, and cold injections into the intestinal canal.

21. Our sensations of heat and cold vary, according to the conducting power of the substances applied to our bodies, and the suddenness of the change in the temperature of the medium in which we are placed.

22. Unaccustomed as we are to immersion in a watery medium, added to its ready conducting power, prevents us from experiencing any very decided sensation of warmth in a bath of less temperature than that of the human body, or at least, of its extremities.

It is a fact familiar to every body, that long exposure to cold, even when not productive of injurious consequences, diminishes the power of forming caloric, or at least produces a sensibility to cold, that makes a reduction of temperature very uncomfortable. It is not, however, so generally known that exposure to a high temperature for a short time, fortifies the body against cold. The Russian goes from a bath of from 120 to 140 deg. of Fahrenheit to enjoy the luxury of rolling himself in the snow; and Acerbi states that Finnish peasants, after a vapour bath of 167 of Fahrenheit, will, for hours, expose themselves naked to an atmosphere 24 deg. below zero. It is to most persons a matter of surprise that they are able to bear these sudden extremes. It is the opinion of the author, that the body derives its power of resisting the influence of cold from the excitement produced by the increased temperature, and that it is only after the system begins to experience the indirect debility resulting from heat, that a sudden change of temperature produces injurious consequences. This opinion we have no doubt is correct, and is worth attending to; and hence it will follow, as an important practical maxim, that while the habitual use of the cold bath will fortify the constitution against cold, the use of the warm bath will best protect against occasional exposure.

Chapter III., on the *Division of Baths*. Individual peculiarities will lead them to ascribe the appellation of cold, tepid, warm or hot, to baths of very different temperatures. Dr. Bell states that a bath below 70 deg. will produce a continued sensation of cold upon all persons, and under all circumstances. A bath ranging between 74 and 82, will be cool, producing a momentary shock, which will probably be followed by a sensation of warmth. We may take 95 deg. as the medium temperature of the warm bath, and by allowing 3 deg. above and below this we will have sufficient room for adaptation to every variety of individual peculiarity.

Vapor baths may be divided into the *moist* or *dry*, and into the *simple* and *medicated*.

Chapter IV. is appropriated to the *cold bath*. The cold bath, the author contends very strongly, is debilitating. He infers this from the symptoms which succeed its application; 1, the constriction and diminished circulation of the skin; 2, the effects upon the internal organs—the lungs, digestive organs, and the brain.

"First then, as to the manner in which the lungs are affected. A warm, and, still more, a hot vapor given out during respiration indicates an active, and in the latter case, highly excited state of the pulmonary circulation, and especially of the capillaries of the lining mucous membrane. Now, if a person, whose lungs are in this state of strong functional exercise, goes into a cold bath, we discover very speedily that the air which is expired is no longer hot or even near so warm as in common, nor so abundant in vapor. In other words, the air taken into the lungs is returned without undergoing changes to the same extent as before the cold immersion; and this is direct evidence of the diminished activity of the pulmonary circulation, and of the secreting function of the respiratory mucous surface, which latter is in fact similarly, though not to the same extent, affected as the skin. Secondly, as to what transpires in the digestive canal; the changes in it are tolerably well represented by the corresponding alterations of appearance in the tongue and lining membrane of the mouth and fauces. It is well known, that when the stomach is highly excited, irritated, or inflamed, the amount of blood circulating through, and contained in its vessels, is greater than before: there is a sensation of heat in the parts, and thirst; the mouth is dry and parched, and the tongue is in the same state, and, in general, preternaturally red. Let a person thus suffering, use the cold bath, and what results? The mouth loses much of its dryness, the tongue much of its redness, if it do not become actually pale, thirst is no longer felt and the sensation of inward heat complained of, as well in the stomach as in the chest, has disappeared. Surely these two series of phenomena in the pulmonary and digestive apparatus, for changes similar to those above described in the state of the stomach, take place also in the course of the intestinal canal, afford no evidence of an unusual determination of blood to the organs of which they consist. On the contrary, we have an intimate conviction, from the feelings and symptoms, that there is now less blood, and a less active circulation, as well as diminished sensibility in these parts, such as we know there is in the skin. If, in the third place, we ask whether the paleness of the face, and the obviously diminished activity of the external vessels of the neck and head be replaced, in the cold bath, by an accumulation of blood in the brain, we cannot, on examination, but answer in the negative. The functions of the brain are vigorous in proportion to the amount of blood distributed to this organ; and it is only when the supply is excessive that the mental faculties, for a while preternaturally active, become perturbed and weakened, as in the state preceding delirium and apoplexy, and pending these maladies. But no symptoms analogous to these are discovered on immersion in the cold bath, and when the presumed determination of blood to the brain is supposed to exist. From the first application of the cold fluid, there follows impaired mental vivacity; the person feels dull, has his range of ideas limited, and perceptions blunted; he is torpid; but it is the torpor of gradually abated cerebral circulation, unaccompanied by those sensations of fulness, and singing in the ears, which would be caused by undue determination of blood to the part. Restoration of the accustomed activity of the faculties and senses generally, in the case of the cold bath, is performed by means the very opposite of those which we would have recourse to where undue determination of blood existed. External warmth, and a mild, stimulating drink, are sufficient to relieve the torpor from cold; they would aggravate the state of a brain, when undue determination of blood has been the cause."

Dr. Bell further supports his opinion 3. From the diminution of muscular power; and 4, the diminished secretion of animal heat. The glow of heat which healthy persons experience immediately after coming out of a cold bath, he asserts is "merely relative to the cold medium in which the body has been immersed a few minutes before," and is in conformity with a very general law of the animal economy, viz. that the abstraction of a stimulus renders the body more sensible to its influence when it is restored. There is, however, at this time, in reality, no increase of the animal heat, on the contrary, the thermometer indicates the temperature to be below the standard of health.

This opinion, he thinks, is still further strengthened by the success of its remedial application,—it being given with advantage in diseases of increased action, and contra-indicated when the powers of the constitution are impaired, and the heat of the body below the healthy standard.

In this mode of reasoning there certainly appears, to us, to be great inaccuracy. It is very true that heat is so essential to the existence of animal life, that any considerable and permanent reduction of the temperature must be fatal. So long, however, as the body is not exposed to such a degree of cold as to carry off the animal heat more rapidly than it can be generated, the sensation of cold is undoubtedly a stimulant. If evidence of this is wanting, it is to be found in the increased capacity to sustain labor and fatigue, and in the florid complexion of those who are exposed to a degree of cold which the powers of the constitution, invigorated by exercise, are enabled to sustain. The body of a healthy man retains for hours the same temperature, in an atmosphere varying at least 200 deg. To what are we to attribute this increased evolution of animal heat, if it does not arise from the stimulating effect of cold. The range of temperature under which the powers of life will continue to resist the pernicious effects of cold; or in other words, under which cold will continue to excite the powers of life to more vigorous exertion, will depend upon the strength of the constitution, upon habit, upon the absence of any present cause of debility, &c.; of course it will vary greatly in different individuals, and in the same individuals under different circumstances. That it really does possess this power, is proved by the fact, that diseases unquestionably dependent upon debility, such as chlorosis, obstinate intermittents, &c., are every day cured by it.

With the views which the author entertains of the *modus operandi* of the cold bath, he of course limits its application almost to diseases which are accompanied with febrile action. He recommends it in those persons who are affected with nervous irritability, accompanied with increased sensibility to atmospheric vicissitudes, but not dependent upon visceral disease. Under these circumstances, the limited use of the cold bath, if the circulation be tolerably active, and the skin not habitually cold, will "blunt the acuteness of sensation by the sedative action of the cold on the nerves," and by increasing the density if not the actual thickness of the skin, cause it to "transmit less readily the impressions of cold and heat." Regular and active exercise in such habits will efficiently co-operate with the use of bathing.

In that kind of nervousness which proceeds from dyspepsia, chronic disease of the liver, or protracted intellectual efforts with late hours, and is accompanied with headache, palpitations, the system is too much enfeebled to bear the sedative action of cold.

Cold bathing he asserts to be useful in persons of sedentary habits, in whom the skin is habitually hot and dry. Its good effects are assisted by moderate exercise, and especially by the use of the flesh brush.

It is not less useful to those who, notwithstanding the enjoyment of good health, are liable to colds, rheumatic and pleuritic affections, &c. The cold both checks the disposition to perspiration, and consequently removes the source of that debility which produces the susceptibility to the causes of disease He denies that the cold here can be considered as a stimulant because its "beneficial operation is evinced at a time when no stimulus or tonic is admissible, and in habits sanguine and plethoric, on whom nearly similar effects would be produced by a moderate bleeding and reduction of the usual quantity of food with diluent drinks." As fallacious a mode of reasoning as can well be conceived of. How often do we see one tissue or one set of organs laboring under debility while another is preternaturally excited. In such cases health may sometimes be restored either by reducing the system, or by applying an appropriate stimulus to the local disease; although in general the combined agency of the two classes of remedies is necessary. We do not say that this is strictly a parallel case, for the question is too complicated to admit of being settled in a very decided and positive manner, but such a solution has at least many probabilities in its favor.

The great field for the cold bath, according to Dr. B. is in the cure of febrile diseases. This practice, with the time and circumstances under which it is applicable, and the proper precautions under which it is to be used, is fully described by authors, in the hands of every physician. It has not, however, by any means received the attention it deserves from the profession at large. We shall not dwell further upon this part of his practice than to give his remarks upon its use in hemorrhage of the lungs.

"Physicians, participating in the notions of the vulgar, that the blood was driven to the internal organs by the application of cold to the skin, were long deterred from the free use of the cold bath in *hemorrhages*. Popular experience had very early shown the good effects of cold applications in bleeding from the nose,—and ought to have been sufficient to expose the fallacy of this hypothesis. Still, the extended use of the remedy to other forms of internal bleeding was exceedingly slow, and looked upon as hazardous in the extreme. Reasoning from the obvious phenomena produced by cold bathing which I have already detailed, we cannot, *a priori* fail to satisfy ourselves, that, consentaneously with the diminished action and temporary torpor of the skin, are similar states of the mucous membrane of the nose, lungs, stomach, intestines, bladder and uterus, on which, respectively, take place epistaxis, hemoptysis, hematemesis, mælena, hemorrhoids, hematuria, and menorrhagia.

In *hemoptysis* or spitting of blood from the lungs, the cold bath, so long deemed a hazardous application, has been tried by several distinguished practitioners with the best effects. The knowledge of its efficacy in this case has been gained probably in an empirical manner—if we consider the erroneous notions regarding the operation of cold on the internal organs. But all doubt ceases by our observing that, simultaneously with the torpor of the capillaries of the skin, induced by the cold bath, is that of the minute vessels of the pulmonary mucous membrane. The collapse of the extremities of the blood-vessels causes a necessary coagulum and a consequent mechanical obstruction to the flow of blood: there is, besides quiescence of the nerves—diminished or abolished sensation, and less afflux of blood to the part. It is obvious, however, that for the cold bath or ablution to be of marked benefit, it must be of so low a temperature and continued so long, as that it may exert a permanently depressing action on the hitherto morbidly excited capillaries of the lungs. If the remedy be of such short continuance to the skin as merely to suspend, without directly lessening the function of calorification in the part, and its vascular excitement, the reaction will be considerable, and the capillaries more distended than before. I have directed cloths, fresh dipped in well water, of 52 deg. Fahrenheit, to be applied to the chest of a patient afflicted with hemoptysis, and have witnessed the good effects of cold in arresting the flow of blood without any accompanying or subsequent inconvenience. Others have had sheets wet with cold water rolled round the chest. Giannini, among other cases, cites the one of a young man seized with spitting of blood, whom he caused to be immersed, for about a minute at a time, in water made colder by the addition to it of ice; this operation was repeated three times in the twenty-four hours, and with a speedy and happy result. He very properly directs a preference to be given to the period of the fever, and of the flush accompanying the discharge of blood. But we need not necessarily wait for or expect always the cutaneous excitement. We must depress, beyond what is pleasurable or any ways comfortable, the cutaneous capillary tissue, in order to act sympathetically on the pulmonary mucous capillaries from which the blood is discharged. If by frequent returns of the hemorrhage, the patient have been much enfeebled, we may substitute affusion or ablution, in the manner already advised for immersion—to which Giannini recommends the additional treatment by frequent and copious enemeta of cold water, and cold acidulated drinks. Currie says, that he has found hemoptysis may be stopped by immersing the feet in cold water, and perhaps this may be done still more certainly by a permanent application of cold to the genital parts. He found the most powerful effect to be produced by immersing the body up to the pubes in cold water, a practice which, he adds, he can from experience speak of as often safe and efficacious in this disease.

If any doubts of the fallacy of the common hypothesis, which supposes a driving of the blood from the external to the internal parts, and from the extremities to the trunk, when cold is applied to the skin or to the feet, should exist in the mind of the reader, they ought to be dissipated by the cases mentioned by Currie. Immersion of the feet, or of the lower extremities entire in cold water, should, according to vulgar belief, by repelling the blood from these parts, precipitate it with increased force and quantity on the chest and head, and aggravate rather than benefit in hemoptysis and epistaxis. That very different effects result is at once a proof of the false doctrine so long prevalent on this subject, and evidence of the correctness of the view which I endeavor to inculcate."

These views upon the subject of the similar state of the evacuation in the different tissues of the body is the doctrine that has been uniformly advocated in this Journal. The opinion that the circulatory system, is not an inanimate conduit whose equilibrium is liable to be perpetually deranged, and whose contents, in consequence, are in danger of accumulating in one part at the expense of the rest, but, is intimately united by a common sympathy which has a tendency to cause every part to assume both the kind and degree of morbid action which prevails in any one part, appears to us to be the view that is best supported by attentive observation of the history of diseases. This subject is the more important because it is not, as are many of the opinions which divide the medical world at the present day, a question destitute of practical importance, but has now, and has always had an important influence upon the practice of medicine.

The cold bath has been used with advantage in many convulsive diseases, as in chorea, epilepsy, &c. But only when some degree of inflammatory action was present.

*Chapter* IV. is devoted to *sea-bathing*. Among other grounds of difference between the effect of bathing in the sea, and in the common cold bath, the most important are "the slower evaporation of a saline than of a simple aqueous fluid," and, "the incrustation on the skin of saline particles, and the consequent stimulus which results therefrom, when the body is subjected to the common friction of the apparel." It is a fact well known to many persons, and abundantly attested by navigators, that persons exposed to the inclemency of the weather, suffer less from being drenched with sea-water than from being soaked in the rain. Dr. Currie attributes this to the stimulating influence of the salt held in solution. Dr. Bell says that any influence of this kind is imaginary, or if there be any, it is to be sought for in the slower evaporation of the salt water, and the consequent less degree of cold produced by it. The stimulus of the salt is produced, he thinks, only from the deposition upon the skin after it is dried. Upon this subject we are much rather inclined to adopt the opinion of Dr. Currie. No individual, we think, who has been accustomed to recommend the warm salt bath, can have failed to remark the much greater length of time that immersion in it can be borne, a circumstance that can be explained only by the supposition that, while still in solution, it exerts a stimulus upon the skin very different from the relaxing influence of the warm bath.

The influence of sea-bathing upon the animal economy is dependent upon many other circumstances, as climate, season, exposure, and exercise, &c. which serve very much to modify its effects, and for the most part to enhance its value. The general principles, however, for its application are the same.

As to the proper time for bathing, the early part of the day is, perhaps, on the whole, the most appropriate. It should never be resorted to when the heat and action of the body is below par, after being exhausted and debilitated by exercise for several hours, nor after a full meal.

Those who are accustomed to bathing, know well, that the reduction of temperature is much greater when exposed to the air, after leaving the water, than from continued immersion. Those persons, therefore, who have any thing to apprehend from the debilitating effects of the cold bath, would do well to continue in the water for some time, rather than have recourse to repeated immersions. Such persons should dry themselves as speedily as possible and resume their apparel.

After leaving the bath, moderate exercise serves to promote re-action, and to equalize the circulation. If an individual has used the bath at an improper time, or under improper circumstances, and coldness and shivering remain for a considerable time accompanied with languor, the invalid should retire to bed, and endeavor to produce re-action by friction and external warmth, especially to the pit of the stomach, and by drinking some aromatic infusion.

Among the diseases which are relieved by sea-bathing, the author enumerates dyspepsia, hypochondriasis, with their numerous associated ailments, such as sick head-ache and palpitation of the heart, hysteria, chlorosis, chorea, asthma, when not purely of the nervous kind, and slow fever. As a general rule the author thinks it will only be successful in curing these diseases when they are accompanied with a hot dry skin and an accelerated pulse, or when there are daily paroxysms of this kind. Females, with a cold and phlegmatic habit, or exhausted by a prior disease, and who are clear of fever, will only be injured by sea or cold bathing.

For a great variety of useful facts and remarks upon the subject of sea-bathing, we must refer to the author himself, simply remarking, that however valuable it may be as a remedial agent, it is by itself, unless the necessary precautions and subsidiary means are assiduously employed, likely to do very little good, and perhaps is more likely than almost any other agent to be so perverted or misapplied by the invalid as to have a pernicious influence.

*Chapter* VI. is devoted to the warm bath. The range assigned to this bath by the author, as we before remarked, is between 92 and 98 deg. The highest extreme of this range will be grateful to those, the temperature of whose bodies from natural or accidental causes, is below the healthy grade. The lowest and from that to 85 deg. will be the range of that indefinite temperature called tepid or milk-warm, which is best adapted to persons of health, for the purposes of cleanliness, or for relaxation from fatigue.

The author strenuously recommends to the imitation of the inhabitants of the cities of this country the example of those nations, both of ancient and modern times, who established public baths for the convenience of the citizens generally. The salubrity of the practice, the importance of habits of cleanliness, not only to health but to good morals, certainly render it a subject worthy of the consideration of the legislator. Whatever reception a proposition of this kind may meet with from the public, a bath should undoubtedly form a part of the furniture of every family. If the luxury itself were not sufficient to recommend it to universal use, the high recommendation of such men as Franklin and Bacon, and the beneficial influence it exerted in renewing the vigor of their youth at a very advanced age, ought at least to entitle it to consideration.

The most important of the effects of the tepid bath are, to allay irritation, to increase absorption and exhalation—to diminish the frequency and increase the fulness of the pulse. Hence it is useful to relieve fatigue—to procure sleep—to remove a dry and husky state of the skin, and restore its natural secretions. It is useful also in all those diseases which have their seat in morbid sensibility, or obstructed perspiration; mania, convulsions, colic, cholera infantum, dysentery, diarrhœa, croup, catarrh, asthma, diseases of the skin, rheumatism and gout, mercurial disease, &c.

There appears to prevail a very general idea that the warm bath debilitates when continued for any length of time. This effect depends entirely upon the temperature; for if the proper medium be observed in this respect, the bath may be continued for hours without superinducing any debility.

The hot bath is peculiarly applicable where there is great torpor and want of action, either generally or locally. In cases of plethora, and especially where there are symptoms of determination to the head, its use is attended with great danger. The heat of the bath should never transcend that of the human body, or the indirect debility which results from its use, may be productive of serious consequences.

Most persons entertain great apprehensions respecting the consequences of going suddenly from a very warm to a very cold medium. These consequences, however, only result when the heat has been so long continued as to relax and debilitate the system, and impair its power of generating animal heat. If it has not been pushed to this extent, an individual may pass with impunity from the hot to the cold bath, and will be better enabled to resist the cold in consequence of being previously subjected to the hot bath, but the use of the former will obviate many of the unpleasant consequences which some constitutions experience from the use of the latter.

The remainder of the work contains some valuable chapters on the subject of *Vapour Baths*, *Mineral Waters*, *sulphurous* and other *fumigations*, the whole of which we cordially recommend to the consideration of the profession, with a firm persuasion that all who read them will feel themselves liberally repaid by the amusement and benefit they will derive from the perusal.

